# Evaluating the impact on physical inactivity of Together an Active Future, a partnership approach to physical activity promotion. A difference-in-differences study

**DOI:** 10.1136/jech-2023-220891

**Published:** 2023-11-06

**Authors:** Gwilym Owen, Katie Fahy, Benjamin Barr

**Affiliations:** Department of Public Health, Policy and Systems, University of Liverpool, Liverpool, UK

**Keywords:** INEQUALITIES, PUBLIC HEALTH, EXERCISE

## Abstract

**Background:**

Low physical activity is one of the leading causes of ill health in the UK and an important determinant of health inequalities. Little is known about the effectiveness of community-wide interventions to increase physical activity and whether effects differ by demographic groups, including area deprivation and ethnicity.

**Setting:**

6 relatively disadvantaged local authority areas in Lancashire, UK, between 2016 and 2021.

**Methods:**

We conducted a doubly robust difference-in-differences study using a large nationally representative repeated cross-sectional survey to investigate the impact of Together an Active Future (TAAF), an intervention aiming to reduce physical inactivity through a programme of creative engagement, partnership building, training and communication. The primary outcome was physical inactivity (the percentage of the population engaging in less than 30 min physical activity of at least moderate intensity per week).

**Results:**

While inactivity increased during the pandemic, it increased to a lesser extent in the intervention population. TAAF was associated with 2.63 percentage point lower level of physical inactivity (95% CI 0.80 to 4.45) in the intervention group relative to the control group. Subgroup analysis found no evidence of differences in effect between groups defined by deprivation, ethnicity, disability, gender or age.

**Conclusions:**

The study suggests that a programme of creative engagement, partnership building, training and communication can help reduce physical inactivity, potentially mitigating some of the effect of pandemic restrictions. Further monitoring is required to understand the impact of this intervention outside of the pandemic context.

**WHAT IS ALREADY KNOWN ON THIS TOPIC**

- While there is some evidence for the effectiveness of multicomponent community physical activity campaigns, there is limited evidence for large-scale interventions and few studies investigate of the differential effects of such interventions across social groups.

**WHAT THIS STUDY ADDS**

- Our study indicates that a large-scale programme of creative engagement, partnership building, training and communication was effective at moderately reducing physical inactivity and we found no evidence that it had a differential effect across different social groups based on age, gender, ethnicity deprivation or disability.

**HOW THIS STUDY MIGHT AFFECT RESEARCH, PRACTICE OR POLICY**

- The creative partnership approach used in Together an Active Future appears to have been effective and other areas aiming to reduce physical activity are likely to benefit from a similar approach. Additional actions, for example reducing cost-related barriers to physical activity may be needed to reduce inequalities.

## Background

Declining physical activity has a negative impact on both physical^1^ and mental health^2^ and is estimated to cost the NHS £455 million a year.^3^ There is a steep socioeconomic gradient in physical activity in the UK with 76% of men in the highest income quintile achieving recommended physical activity levels compared with only 55% of men in the lowest quintile.^4^ The pandemic has increased physical inactivity and exacerbated these inequalities. Increasing levels of physical activity in more disadvantaged groups could improve overall population health and reduce health inequalities.

There is evidence for the effectiveness of multicomponent community physical activity campaigns, those that involve face-to-face or group sessions, social networks such as buddy schemes and those that involve community members in their design.^5–8^ There is, however, limited evidence of the differential effects of such interventions across social groups and concern that some interventions increase inequalities by benefitting less disadvantaged groups the most.^9^ One systematic review found that interventions designed for specific ethnic groups were more likely to be effective although interventions aimed at people with low socioeconomic status tended to be less effective.^5^ Cost can be a barrier to physical activity and studies have found that interventions reducing the cost of engaging in physical activity have been more effective in more socioeconomically disadvantaged groups than in more affluent groups.^10 11^ This evidence is however from prior to the COVID-19 pandemic and may not reflect the impact of such interventions during a pandemic when there were restrictions on face-to-face contact and social mixing.^12^

A large body of work has looked at the importance of place for health outcomes^13 14^ and as such there has been increasing interest in area-based interventions^15^ for improving health and combatting health inequalities. Area or place-based interventions are one way of moving the focus away from individual behaviour change to changing the context in which individuals make their health behaviour related decisions, which has been argued to have a greater impact at the population level.^16^ The evidence for the effectiveness of area-level health and physical activity interventions is mixed, although the components that make up these approaches can vary considerably.^9 15 17 18^

In 2017, Sport England, the non-departmental public body responsible for growing and developing sport and encouraging physical activity in England, funded a set of 12 locally delivered pilots across different parts of England.^19^ These pilots aimed to transform the delivery of physical activity services locally and were granted a combined £100 million of funding over 4 years. Each pilot took an approach specific to its place, in which sport and activity were designed and delivered to meet the needs and wishes of those living there. Together an Active Future (TAAF) is one of these pilots, based in Lancashire, and received £10 million in funding between 2018 and 2021. The intervention has involved a process of creative engagement, partnership building, training and communication aiming to instigate a differentiated approach expanding opportunities for and participation in physical activity. Activities started in 2018; however, from 2020 the programme had to adapt to the conditions of the pandemic, which for periods of time made face-to-face activities impossible. Restrictions on face-to-face activities were in place for 11 out of 24 months during 2020 and 2021.^12^ TAAF worked closely with community groups during this time to keep people connected, supporting them to re-engage in physical activity when restrictions were lifted.

We therefore investigated the impact of the TAAF scheme on physical inactivity in the North West of England. Our aim was to understand whether the approach mitigated any adverse effects of the COVID-19-related restrictions and to investigate the differential effect across groups defined by age, sex, deprivation, ethnicity and disability.

## Methods

### Setting

The TAAF intervention took place between 2018 and 2021 across six districts in Lancashire, UK, known as Pennine Lancashire (Blackburn with Darwen, Burnley, Hyndburn, Pendle, Rossendale and Ribble Valley), covering a total population of half a million people. Five of these are relative deprived towns, while one is a relatively affluent rural area (Ribble Valley). The TAAF collaboration submitted an application for funding through a competitive process in 2017 and were 1 of 12 local delivery pilots funded by Sport England following this competition.

### The intervention

TAAF is a multicomponent intervention. This has included developing a set of guides, tools and principles for engagement, including a set of design principles and guides for creative engagement.^20^ Using these tools, TAAF has been undergoing a process of engagement aiming at bringing together all the stakeholders in a community who have a role in physical activity delivery including local leaders, organisations and passionate individuals.^21^ Through creative engagement sessions, partnerships have been formed that have led to the development of activities. For example, a partnership between the Department for Work and Pensions and Active Lancashire led to the development of Youth Employment Hubs. Part of the offer supported through TAAF has been Creative Football, an organisation that offers football-based activities for adults with mental health and well-being challenges. The implementation of TAAF reflects the theoretical approach of Sport England that positive change is brought about through systems and interactions with them rather than through specific interventions and organisations.^22^

### Data and measures

We use data from a nationally representative annually repeated cross-sectional survey, the Active Lives Survey^23^ of physical activity and sports participation in England. The fieldwork for the survey is spread throughout the year, giving yearly repeated cross sections of the English population with an annual sample size of close to 200 000. We use data for the first 6 years of the survey between 2016 and 2021. The survey is a ‘push-to-web’ survey that involves postal mailouts designed to encourage participants to complete the survey online. The survey sample is randomly selected from the Royal Mail’s Postal Address File.

Our primary outcome was proportion of respondents in the Active Lives Survey who were physically inactive, as this reflected the stated aim of Sport England’s local delivery approach.^24^ An individual was considered physically inactive if they reported doing less than 30 min of moderate-intensity physical activity per week.^25^ Secondary outcomes included the proportion of respondents reporting more than 30 min participation per week in specific activities, including (1) cycling, (2) walking and (3) sport/gym/fitness activities.

Additional variables included in the analysis were age group (16–24, 25–44, 45–64, 65+), gender (male or female), ethnicity (white, Asian, black or other/mixed), the Index of Multiple Deprivation^26^ (a composite area-level measure of deprivation) in which an individual lives, whether an individual lives in an urban or rural area based on the Office for National Statistics classification^27^ and whether they have a disability, based on reporting in the survey that they had a long-term condition that limited daily activities.

We used data on each individual local authority of residence to indicate whether they lived in the Pennine Lancashire area of the TAAF intervention. For the control group, we included individuals in all other local authorities in the North West of England that were not Sport England local delivery pilots and were not less deprived than the minimum deprivation of the TAAF areas and were not less urban than the minimum for the TAAF local authorities. We limited the control group to the North West as regions across the country were affected very differently by the pandemic and experienced different levels of restrictions; however, the impact within the North West was more similar.^28^ This provided 21 local authorities that contributed to the control group (see online supplemental appendix 1 for details). Three per cent of the data was missing for at least one of the variables used. We considered the three survey years 2016–2018 as the preintervention period and the three survey years 2019–2021 as postintervention. This provided a sample size of 17 327 in the intervention group and 71 286 in the control group for the analysis. Survey weights were derived using a raking procedure^29 30^ so that the weighted samples matched the population distribution across age and deprivation groups within each local authority using population estimates from the Office for National Statistics.^31^ The distribution of the survey sample characteristics is given in online supplemental appendix 2.

10.1136/jech-2023-220891.supp1 Supplementary data

### Analysis

To estimate the average effect of the intervention on levels of physical inactivity across the Pennine Lancashire population, we used difference in differences with a doubly robust estimation procedure.^32^ Difference-in-difference methods compare the average change in an outcome over time in an intervention group to the average change over time for a control group. The difference in these changes can be taken as the average treatment effect of the intervention if a set of assumptions holds, the most important being the parallel trends assumption. The parallel trends assumption requires that in the absence of the intervention, the difference between the intervention and the control group is constant over time.

While it is not possible to be certain whether this assumption holds, we can make it more plausible by conditioning on preintervention covariates in order to balance the intervention and the control group in terms of characteristics that might be related to the evolution of the outcome. To do this, we apply the doubly robust estimation method developed by Sant’Anna and Zhao^32^ that combines two different methods for doing this. The first is a propensity score model which uses logistic regression to estimate the probability of an individual being in the intervention group. From this, inverse probability of treatment weights is calculated and applied in the analysis. The second is a linear regression for the evolution of the outcome variable for the intervention and comparison group in both pretreatment and post-treatment periods. The same set of covariates are used in both models, defined as outlined above, including deprivation, age, gender, ethnicity, urbanicity, disability and the percentage of people who were physically inactive in each of the years prior to the start of the intervention in the individual’s local authority. The models were additionally weighted using survey weights accounting for survey non-response and missing data. This doubly robust difference-in-differences approach only requires the correct specification of either the propensity or the outcome model so is robust to model misspecification, but will be biased if both models are miss-specified.^32^ To investigate whether trends in the outcome were parallel in the intervention and control groups, prior to the intervention, we tested whether there was a difference in trends using a linear regression model with an interaction term between intervention group and year.

To initially visualise the data, we plot the trends in mean inactivity levels from 2016 to 2021 in our intervention and control groups, weighting the sample with the survey response weights and the inverse probability of treatment, derived from the propensity model.^33^ We repeat the same analysis for our secondary outcomes. We further explored heterogeneity of effects by estimating the same models across subgroups including: (1) three age groups (16–24, 25–65, 65+), (2) two levels of deprivation—individuals in the most deprived 50% of areas and the least deprived 50%, (3) people with and without a disability, (4) people from black and minority ethnic groups compared with people of white ethnicity and (5) male and female.

In sensitivity analysis, to investigate the reliance of our results on inverse probability of treatment and survey response weights we repeated our main difference-in-differences model, using neither inverse probability of treatment or survey response weights, without inverse probability of treatment weights but including survey response weights and including inverse probability of treatment weights and no survey weights. To investigate whether change over time in the composition of treatment and control survey samples might bias our results, we repeated the analysis with inverse probability of treatment weights stratified by survey year. All analysis was carried out using the statistical software R V.4.3.0.

## Results

Table 1 shows the distribution of individuals across the demographic variables included in the analysis, comparing the control and intervention groups, after weighting for the inverse probability of treatment. For most variables, the intervention and control groups are comparable, though the intervention areas are slightly less deprived than the control areas and slightly less urban, with a slightly higher proportion of disabled people and people from black and other/mixed ethnicities.

**Table 1:** Comparison between the intervention (TAAF) and control populations after weighting for the inverse probability of treatment

	Control	TAAF	P value
Deprivation quartile 1 (least deprived) (%)	21.7	25.1	<0.001
Deprivation quartile 2	22.7	22.3	
Deprivation quartile 3	22.5	23.9	
Deprivation quartile 4 (most deprived)	33.2	28.7	
Male (%)	44.6	44.5	0.87
Urban (%)	80.5	71.7	<0.001
Inactive in 2018 (%)	26.6	26.5	0.14
Age (mean)	53.5	54.4	<0.001
Black (%)	0.4	0.3	0.002
Asian (%)	2.7	2.7	
Other/mixed ethnicity (%)	1.2	1	
Disabled (%)	21.3	19.8	<0.001

TAAF, Together an Active Future.

**Figure 1:**
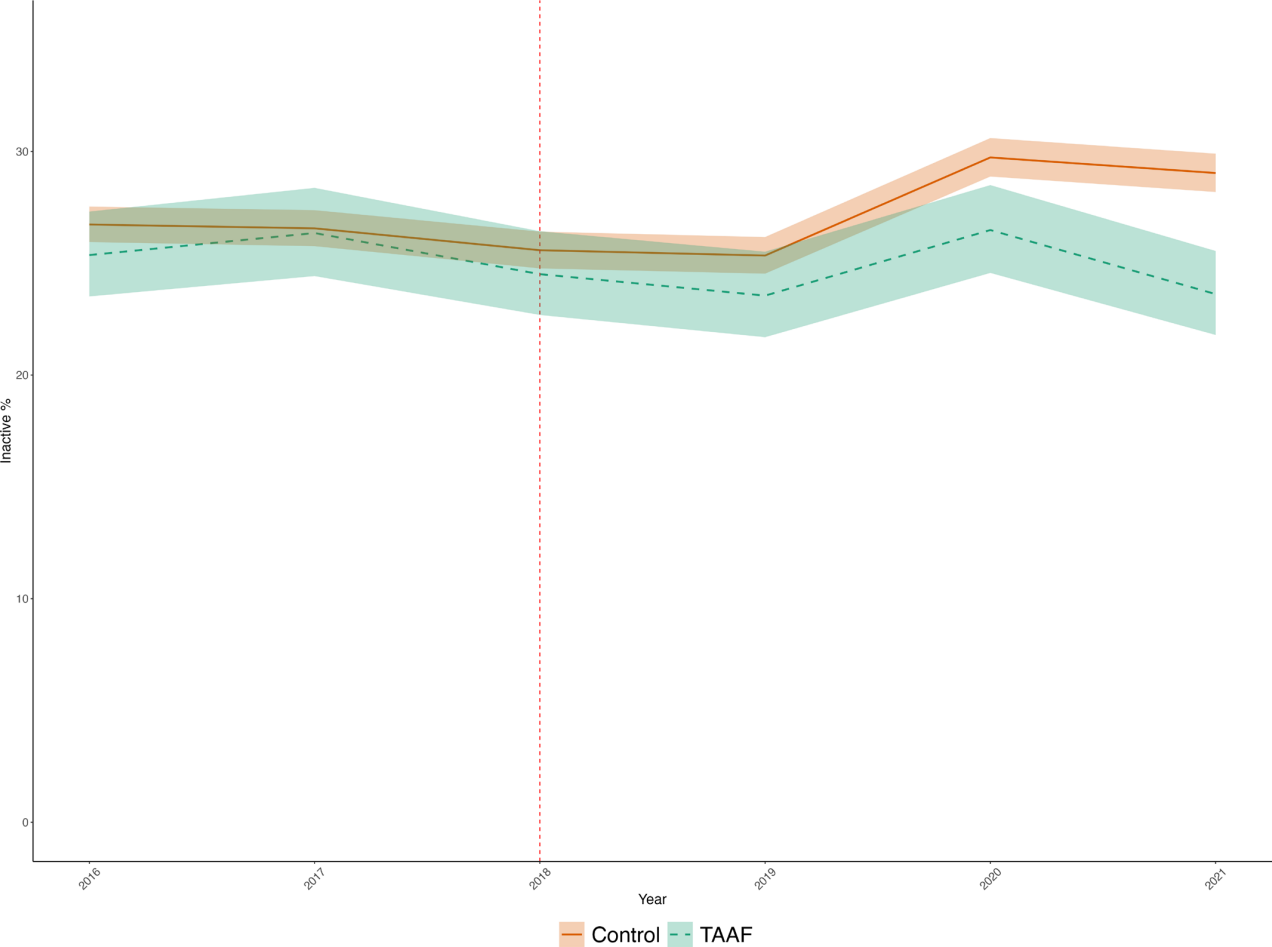
Trend in inactivity in the TAAF areas and in the control group, weighted for the inverse probability of treatment. TAAF, Together an Active Future.

Figure 1 shows the trend in inactivity in the intervention and control groups. Before the intervention, physical inactivity is at a similar level in both groups and declining. In the first year of the intervention (2019), they fall slightly more in intervention areas than in the control group, during the pandemic (2020–2021) inactivity levels increase rapidly; however, this occurs to a lesser extent in the TAAF areas compared with the control. In 2021, inactivity returns to a similar level as 2019 for the intervention group, though the control group does not recover as much with inactivity levels remaining higher than prepandemic. During the preintervention period, there was no statistically significant difference (p=0.9, for group*time interaction) in trends in inactivity between the TAAF areas compared with the control suggesting that the parallel trend assumption was not violated in this analysis.

Table 2 shows the results from the doubly robust difference-in-differences model, indicating the TAAF intervention was associated with a 2.6 percentage point decrease in physical activity, relative to the control group. In other words, we estimate that inactivity would have been 2.6 percentage points greater in the absence of TAAF. Given the adult population in Pennine Lancashire is 456 639, this represented an additional estimated 9100 people being physically active as a result of TAAF, than would have otherwise been the case. When looking at the specific activities affected, it is clear that the greater than expected physical activity in Pennine Lancashire is largely due to increased participation in sport, fitness or gym activities, with an additional 3 per 100 people participating in these activities in Pennine Lancashire than would have been expected in the absence of the intervention.

**Table 2:** Results from doubly robust difference-in-difference model showing reduction in inactivity and increases in participation in specific activities in TAAF areas, relative to the control group

	Estimate	95% CI	
		Lower	Upper
Percentage point reduction in inactivity associated with TAAF.	−2.63	−4.45	−0.80
Percentage point increase in participation in specific activities			
Cycling	−0.24	−1.48	0.99
Walking	−1.46	−3.26	0.34
Sport/fitness activities/gym	3.13	−1.36	4.91
Swimming	0.73	−0.17	1.63

TAAF, Together an Active Future.

**Figure 2:**
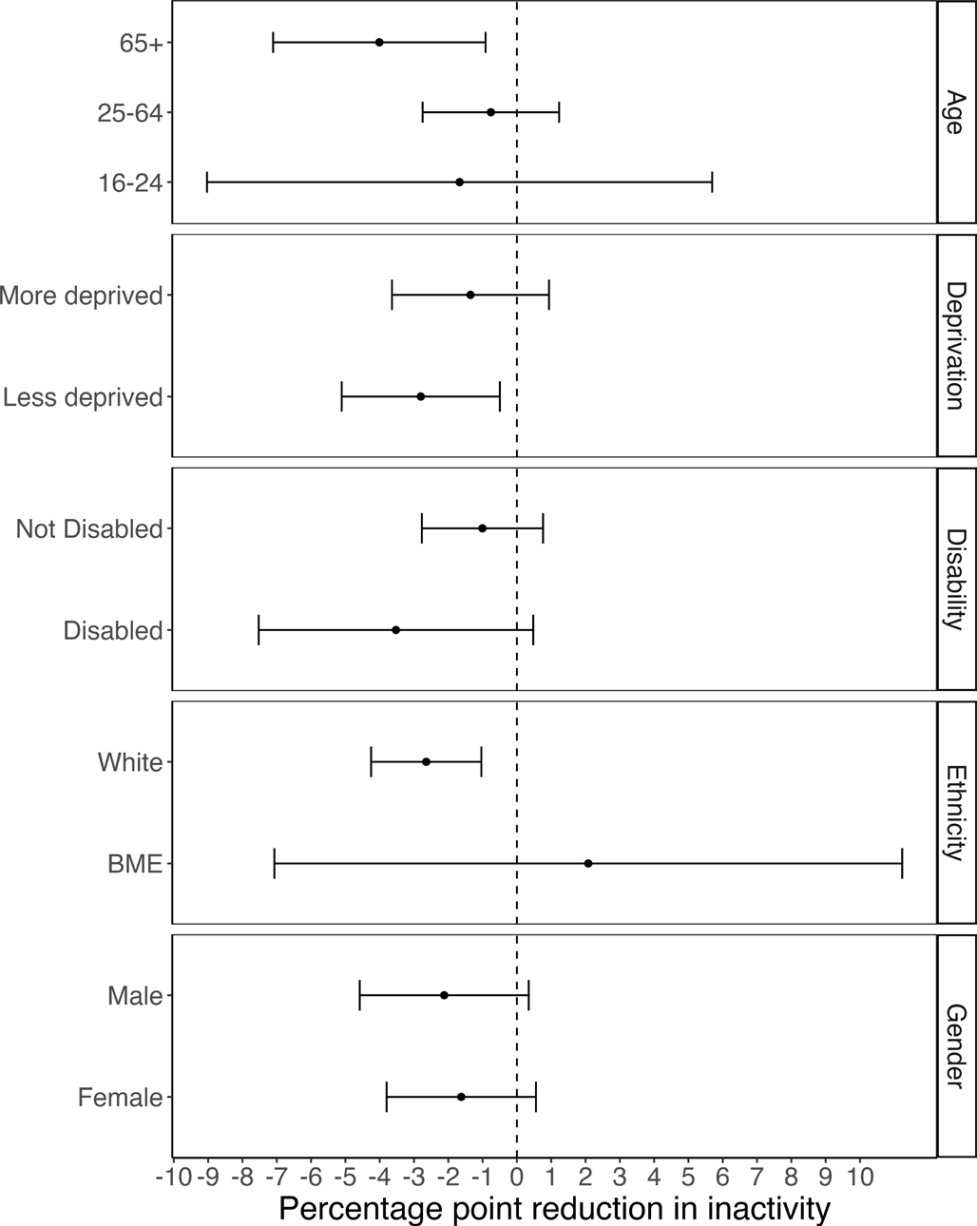
Estimates from doubly robust difference-in-differences model showing percentage point reduction in inactivity associated with TAAF, for subgroups. BME, black and minority ethnic; TAAF, Together an Active Future.

Figure 2 shows the results of subgroup analysis. Due to the large amounts of uncertainty due to small numbers in subgroups, it is difficult to draw any firm conclusions. While the point estimates are greater in older age groups, compared with younger age groups, in less deprived areas compared with more deprived areas, and among people of white ethnicity compared with people from black and minority ethnicities, the CIs all overlap indicating we find no strong evidence of a difference in effect between these subgroups.

Sensitivity analysis indicated that our results did not change markedly when different weighting strategies were used (see online supplemental appendix 3).

## Discussion

We found that the introduction of the TAAF scheme, which focused on building partnerships to promote community-based physical activity, led to lower levels of inactivity than would have been expected in the absence of the intervention. This seems to have been due to increased engagement in sport and fitness activities in the intervention population. The size of the effect is relatively small, equating to around 9000 additional people engaging in some activity, out of a population of around half a million.

National agencies such as Sport England aim to support place-based initiatives such as TAAF that are tailored to the needs of different communities, rather than prescribing specific approaches. It is believed that such approaches will be more effective and sustained as they are better adapted to local needs. This presents a challenge given that the evidence base tends to focus on narrowly defined interventions, rather than such complex interventions that aim to change social norms.^34^ While there is evidence for the effectiveness for some components of community-based approaches such as group sessions, social networks such as buddy schemes and those that involve community members in their design,^5–8^ there is very limited evidence for interventions such as TAAF that aim to initiate change within a complex system by empowering organisations and communities to make connections and changes that eventually lead to actions that increase physical activity. Our study indicates that it is possible for such an approach to have population-level effects.

A large part of the intervention took place during the COVID-19 pandemic when patterns of physical activity were very different.^12^ Furthermore, the intervention itself had to be adapted, as face-to-face contacts where not possible during some periods. For these reasons, we cannot assume that our results will generalise outside of the pandemic context and further monitoring is required to understand the impact of the intervention on postpandemic conditions. Nevertheless, we might expect the intervention approach of empowering organisations and communities to be less successful during the pandemic, so the fact that population-level effects are detectable even during this time is promising.

Interventions such as TAAF aim to reduce health inequalities by targeting more disadvantaged populations. There is also a risk that such approaches engage with relatively small sections of society, benefiting those social groups that already have existing assets. We found no evidence of a difference in effect across social groups. An evaluation of a previous intervention in one of the TAAF local authorities that focused on providing cost-free access to leisure facilities^10^ found a similar size of impact on physical activity levels. That intervention however did lead to greater increases in physical activity in the most disadvantaged groups compared with less disadvantaged groups. Our study therefore provides evidence of a population impact of approaches such as TAAF, while highlighting the need to address other barriers such as cost to have an impact on inequalities in physical activity.

### Strengths and limitations

Our study has a number of strengths. First, by using a large consistent survey dataset, we were able to apply recent innovations in difference-in-differences design that combine the benefits of inverse probability weighting to adjust for selection bias into treatment groups and difference-in-difference methods that account for time-invariant observed and unobserved confounders. This doubly robust approach overcomes several potential biases experienced by other quasi-experimental approaches. Second, our analysis is strengthened by finding plausible results across specific areas of participation. Finding that the decreases in inactivity are due to increases in engagement in sport is consistent with the activities that have been supported through TAAF.

A number of limitations however remain. Self-reported physical activity in surveys will be subject to biases in reporting and recall. Validation studies of self-reported questionnaires have shown inconsistent results when compared with more robust methods.^35^ The Active Lives Survey is a telephone- and internet-based survey with a low response rate, which may affect the validity and reliability of the data. Weighting analysis by survey response weights, as outlined, however, will account for response bias related to age and deprivation. While our methods are robust to some forms of observed and unobserved confounding, shocks that differentially affected the intervention and control groups could bias our results. Given the period of study occurs during a pandemic, one concern is that the pandemic may have had a different effect on control and intervention groups, for reasons that were not connected to the intervention. We have to some extent minimised the chances of that by restricting the control sample to the North West. Finally, the survey has a repeated cross-sectional structure rather than a longitudinal design which means that changes in the composition of the sample may affect our results, although this is to some extent accounted for in the survey weights.

### Implications for policy

Our study indicates that a community-based approach focused on building partnerships for promoting physical activity can produce population-level benefits. There is an urgent need to ensure that the huge decrease in physical activity that occurred during the pandemic does not become a long-running legacy, with persisting public health consequences. Our study indicates that the approach taken in Sport England’s local delivery pilot in Pennine Lancashire could contribute to that recovery. It is crucial however that such programmes maintain a focus on inequalities. That means monitoring uptake and assessing impact across equity dimensions including by ethnicity, socioeconomic status and disability. Importantly, they need to address barriers that are differentially experienced by more disadvantaged groups, such as cost.

## Data Availability

Data may be obtained from a third party and are not publicly available. Data are available through an end user licence from the UK Data Service.
