# Supplementary material for: Evaluating the impact on physical inactivity of Together an Active Future, a partnership approach to physical activity promotion. A difference-in-differences study
Source: J Epidemiol Community Health. 2023 Nov 6;78(2):115–20. doi: 10.1136/jech-2023-220891 (PMC10850728; doi:10.1136/jech-2023-220891)
Supplement: Supplementary data [file jech-2023-220891supp001.pdf]

Appendix 1. Local Authorities included in the control group.

Halton
Warrington
Blackpool
Cheshire East
Cheshire West and Chester
Barrow-in-Furness
Carlisle
Copeland
South Lakeland
Chorley
Fylde
Lancaster
Preston
South Ribble
West Lancashire
Wyre
Knowsley
Liverpool
St. Helens
Sefton
Wirral

Appendix 2. Description of the sample

Year	2016	2017	2018	2019	2020	2021
n	15637	15140	14534	14416	14410	14476
IMD (%)						
Least deprived quartile	3355 (21.5)	3259 (21.5)	2818 (19.4)	2863 (19.9)	2533 (17.6)	4377 (30.2)
2nd least deprived quartile	3595 (23.0)	3623 (23.9)	3363 (23.1)	3215 (22.3)	3415 (23.7)	3056 (21.1)
2nd most deprived quartile	3351 (21.4)	3166 (20.9)	3229 (22.2)	3165 (22.0)	3160 (21.9)	3697 (25.5)
Most deprived quartile	5336 (34.1)	5092 (33.6)	5124 (35.3)	5173 (35.9)	5302 (36.8)	3346 (23.1)
Male (%)	6896 (44.1)	6744 (44.5)	6473 (44.5)	6444 (44.7)	6397 (44.4)	6583 (45.5)
Urban (%)	12608 (80.6)	12161 (80.3)	11747 (80.8)	11642 (80.8)	11679 (81.0)	11706 (80.9)
AGE (mean (SD))	53.23 (17.28)	53.55 (17.32)	53.32 (17.44)	53.00 (17.32)	53.28 (17.38)	53.89 (17.38)
Ethnicity (%)						
Asian	440 (2.9)	464 (3.2)	453 (3.2)	502 (3.6)	506 (3.6)	476 (3.4)
Black	52 (0.3)	64 (0.4)	63 (0.4)	67 (0.5)	61 (0.4)	72 (0.5)
Other/Mixed	156 (1.0)	171 (1.2)	183 (1.3)	167 (1.2)	181 (1.3)	203 (1.4)
White	14578 (95.7)	14011 (95.2)	13397 (95.0)	13238 (94.7)	13244 (94.7)	13275 (94.6)
Disability (%)	2987 (20.1)	3026 (21.0)	2812 (20.2)	2885 (21.0)	2918 (21.4)	3064 (22.3)

Appendix 3. Sensitivity analysis.

Table A3. Percentage point reduction in inactivity associated with TAAF for 5 models with alternative combinations of (1) inverse probability of treatment weights (IPTW) and (2) survey weights. Model 5 uses IPTW that are stratified by survey year.

			**95% CI**	
	Model	**Estimate**	**Lower**	**Upper**
1	IPTW+Survey weights	-2.63	-4.45	-0.80
2	NO IPTW + NO Survey weights	-1.95	-3.42	-0.47
3	IPTW + NO Survey weights	-2.14	-3.77	-0.51
4	NO IPTW + Survey weights	-2.11	-3.72	-0.49
5	Annual IPTW	-2.28	-3.99	-0.57
